# A Very Remarkable Case of Valvular Disease of the Heart, Resulting in Absolute Death of a Portion of the Inferior Extremities

**Published:** 1857-04

**Authors:** F. R. Payne

**Affiliations:** Marshall, Ill.


ARTICLE II.—*A very remarkable Case of Valvular Disease of the Heart, resulting in absolute Death of a Portion of the Inferior Extremities.* By F. R. PAYNE, Marshall, Ill.

It will undoubtedly be interesting to the professional readers of your *Journal* to have a brief account of the above wonderful case. All who saw it were struck with amazement at its unparalleled peculiarities. We will endeavor to give a brief and truthful history of the case without referring particularly to the treatment, from the fact that there was nothing peculiar in it, and I was well satisfied from the commencement that it could only be palliative, and the disease must inevitably terminate in death.

Mrs. Margaret English, aged fifty-five years, living on a farm; she was rather corpulent. Some years ago she was thrown from a horse, and since that time has complained of palpitation of the heart. Last fall she had a violent attack of bilious fever. During that time I visited her daily; her pulse was intermittent during the entire progress of the disease; she frequently had great dyspnœa. In eight or ten days the fever subsided, and she was again enabled to attend to the ordinary duties of the house, but was troubled with what she called 'palpitation.'

*Feb. 2d*—She is now suffering with excessive pain in her legs; they are very cold; there is no discoloration of the skin; pulse not very frequent, but intermittent; the rational and physical symptoms clearly indicated disease of the heart. Tongue partially covered with a brown fur; bowels costive. Ordered blue mass to be followed with powders of quinine and opium. Mustard and stimulating liniments to the cold and painful limbs. After this, veleriated tinct. ammonia three times a day in tea-spoonful doses.

——*4th*—Called in great haste. She has felt better until to-day; but now has a return of pain in her legs, suffering almost intolerable; some heat in the limbs; veins congested, and blood very dark; pulse feeble and intermittent; distressing dyspnœa at times.

——*5th*—Some less pain in her legs; action of the heart the same; there are many red spots on her legs, but no heat, rather an icy coldness; partial loss of sensation in the toes.

——*6th*—The symptoms about the same, except the red spots which are now confluent; toes shrunken, dry, and lifeless.

——*7th*—There is a general red appearance of the skin of the legs; the toes are dry, hard, and present a reddish transparency—they are absolutely *dead*, dry, stiff, and transparent; and there is but little sensation in the deep red skin below the knee; she has some appetite and feels easy.

*Feb. 8th*—Complains of excessive heat in her limbs, but they are icy cold to the touch; toes as before; intolerable pain in the large muscles of her legs, and feel like they are firmly compressed by an iron band. The skin presents a dark reddish appearance, and is absolutely dead, hard, dry, and contracted, except a small patch on the top of each foot; some red spots above the knee; action of the heart about the same.

——*9th*—She is more comfortable; pulse the same; the red spots above the knee are closer and more diffused, the skin beneath them is perfectly dead, dry, and contracted.

From this time until the 24th, there was but little variation in the symptoms and appearance of the parts. I visited her every day, two or three times. Drs. H. R. Payne, and L. C. Churchill carefully examined the case. There was but one opinion in relation to the nature of the disease. The physical and rational symptoms seem to clearly indicate valvular disease of the heart, and they agreed with me that the treatment could only be palliative.

——*25th*—The skin on the upper part of right foot has sloughed, but this process has not extended beyond the limits of the patch that has retained to some extent its normal color. No sensation in the legs, except in the large muscles; red spots begin to appear on the back of her hands; action of the heart very feeble and intermittent; bowels very torpid.

——*26th*—No more sloughing; the limbs begin to shrink; the dead skin is wrinkled, and seems partially detached from the muscles—when pressed upon, we have a crepitating sound; severe pain in the region of the heart; she rests better when propped up in bed; there has been no mental derangement during her sickness. The death of the skin did not extend after this date, but the affected limbs continued to shrink. It is now utterly impossible to get an action on her bowels by ordinary means.

She died on the second day of March.

This was truly a wonderful case, and we would be pleased to have the opinion of yourself and others in relation to its peculiarities. The case clearly proves that disease of the heart may terminate in death of the limbs—a fact that has never been noticed by any medical writer with whom we are acquainted. I regret that I could not obtain a post mortem.

